# Alveolar Exostosis

**Published:** 1843-09

**Authors:** S. M. Shepherd

**Affiliations:** Petersburg, Va.


### ARTICLE VI.

### *Alveolar Exostosis.* By S. M. SHEPHERD, D. D. S. of Petersburg, Va.

MRS. H. a lady residing in this town, about twenty-five years of age, and whose health has been uniformly good, called on me about three months ago, saying that she had "something growing in her mouth," which gave her no pain, but from the size which it had attained, was a considerable annoyance. Upon examination I found that the first inferior molar tooth of the right side had been removed by decay, and there was a growth of flesh occupying the entire space. This flesh had risen up even with the grinding surfaces of the teeth, and I suppose would have been much higher if the teeth above had not met upon it and kept it down. It had also spread out on each side something like half an inch, so that the outer portion gave the jaw precisely the appearance of a badly swollen gum from tooth-ache.

Upon inquiry I learned that it had been in progress about three years, during which time it had acquired great solidity. Pressure upon it with the end of the finger was very similar to that of a child's gum over a molar tooth just before its eruption. Its immediate removal was determined upon. Owing to the situation and size of the excrescence, I thought it best to divide it in the centre, and upon a line with the alveolar ridge. This I did with a common gum-lancet without meeting with any obstruction. I then attempted to cut away the inner portion, when to my surprise, the instrument after passing through the surface met a firm resistance—so that it required a considerable effort with a strong blade of a knife to divide it from the alveolus. This substance proved to be a new growth of bone, quite cellular in its structure, and resembled the inner portion of the vertebra. This bone grew up from each edge of the alveolus in two distinct portions—each one being about the size and shape of a pea; which fact I ascertained by removing the flesh or skin from one of them which I preserved in alcohol. After removing the excrescence I searched in vain for the roots of the tooth which I suppose produced it; the entire destruction of which must have been completed previous to the excision.

The operation was followed by considerable hemorrhage, but was not attended with much pain. The fleshy portion of the protuberance being perfectly insensible. By this simple operation, a perfect cure was soon effected.

